# Direct Look from a Predator Shortens the Risk-Assessment Time by Prey

**DOI:** 10.1371/journal.pone.0064977

**Published:** 2013-06-05

**Authors:** Sang-im Lee, Soyun Hwang, Young-eun Joe, Hyun-kyung Cha, Gun-ho Joo, Hyeon-jeong Lee, Ji-won Kim, Piotr G. Jablonski

**Affiliations:** 1 Institute of Advanced Machinery and Design, Seoul National University, Seoul, Korea; 2 School of Biological Sciences, Seoul National University, Seoul, Korea; 3 Centre for Ecological Studies, Polish Academy of Sciences, Dziekanow Lesny, Lomianki, Poland; University of Sussex, United Kingdom

## Abstract

Decision making process is an important component of information use by animals and has already been studied in natural situations. Decision making takes time, which is expressed as a cost in evolutionary explanations of decision making abilities of animals. However, the duration of information assessment and decision making process has not been measured in a natural situation. Here, we use responses of wild magpies (*Pica pica*) to predictably approaching humans to demonstrate that, regardless of whether the bird perceived high (decided to fly away) or low (resumed foraging) threat level, the bird assessed the situation faster when approaching humans looked directly at it than when the humans were not directly looking at it. This indicates that prey is able to extract more information about the predator’s intentions and to respond sooner when the predator is continuously (“intently”) looking at the prey. The results generally illustrate how an increase of information available to an individual leads to a shorter assessment and decision making process, confirming one of central tenets of psychology of information use in a wild bird species in its natural habitat.

## Introduction

Processes of assessment and decision making [1]–[4] have been studied in natural situations in various contexts including mate choice [5]–[7], habitat choice [8]–[10] and foraging [11]–[13]. Prey escape responses to predators also provide opportunities to study the assessment (predation risk assessment) and the consequent decision-making processes. The studies of prey escape reactions are dominated by cost-benefit analyses in the tradition of behavioral ecology (e.g. [14]–[16]), or by proximate analyses of simple and fast escape responses (e.g. [17]–[20]) motivated by neurophysiological and neuroethological research. Some studies attempted to combine these two approaches trying to determine how simple responses (unlike more complex cognitive processes of assessment) of prey can lead to optimization of prey survival (e.g. [21]–[23]).

The studies of prey decision making based on more complex assessment processes (cognitive processes rather than simple mechanisms such like prey escape responses to looming stimuli [17]–[23]) have mostly focused on determining the assessment rules (e.g. [24]–[27]), perhaps because the assessment rules are at the center of attention in neurobiology [28], evolutionary psychology [29] or cognitive ecology [30]. They are also important for adaptive interpretations of prey flight initiation distances (e.g. [27], [31]–[34]). The costs of decision making process, such as the time required for risk assessment, are important for the theory of information use in ecology [2]. However, the time required for assessment and its influence on decision making process have not been determined in natural situations and have not been explicitly incorporated in the classical theoretical models of escape behavior (e.g. [14], [16], [33]).

The process of assessment and decision making requires time [35], and it may be shorter or longer depending on the factors that affect the amount of information available to the prey [2], [6], [36]. Some of these factors may be the very same factors that normally indicate higher predation risk or stress in general. Direct gaze, looking at the prey, is one of them. For example, prey seem to treat the direct gaze or direct “looking” of the predators as an indicator of higher predation risk [37]–[43]. However, it has been documented that gaze, face and head, as well as their movements, may also contain important clues that may allow humans or computer algorithms to predict the actions of the subject [44]–[47] regardless of how threatening the action may be. These findings suggest that gaze and face may provide crucial information to the prey about predator’s future actions. If animals use this extra information associated with the predator looking at them in their risk assessment, this may lead to the shorter assessment duration when a predator looks directly at the prey (regardless of the outcome of the assessment). This idea has never been tested because previous experiments were based on the notion that direct look at the prey, as well as the face/head orientation, solely indicate higher predation risk to the prey and that prey adjusts its behavior accordingly. Furthermore, the previous experiments usually resulted in only one behavioral outcome: fleeing from the predator (e.g. [48]). In this situation it is impossible to study the duration of assessment that is independent from the outcome of assessment.

Here, we follow Blumenstein and Bouskila [25]’s suggestion that “observing the resultant behavior may permit inferences about assessment only when there are different behavioral responses to the stimuli”. Although originally this referred only to the inferences about assessment rules rather than costs or durations of this process, we apply their idea to test the prediction that direct look by a predator at a prey shortens the risk assessment and decision-making processes in the prey regardless of the level of predation risk estimated by the prey. We used a situation where both types of behaviors were observed in the urban population of the Eurasian Magpie, *Pica pica*: the behaviors that indicate high perceived predation risk (flee from an approaching predator), and those that indicate low perceived predation risk (ignore predator’s approach). Birds, like magpies, show behaviors that indicate when they became alert and therefore it is possible to measure the assessment time between the moment of becoming alert and the initiation of behavioral response. If the direct look of the predator provides the prey with some additional information useful in the risk assessment, then the assessment process should be shorter [6], [36] regardless of whether the outcome of the assessment indicates high or low risk, that is regardless of whether a bird flies away or resumes foraging. On the other hand, if the effect of approaching humans on the bird’s timing of behavioral response is mostly due to variation in the perceived level of threat [42] or stress [55], rather than due to the amount of information available to them, then a bird whose behavioral response indicates higher perceived threat/stress level should take the decision sooner than a bird whose behavioral response indicates lower level of perceived threat/stress. Hence, the bird who flew away should take the decision sooner than the bird who decided to resume foraging.

In this study, we compare the effect of gaze on the duration of the assessment, between situations when a bird decided to fly away (outcome of an apparent assessment of high threat level) and the situations when a bird decided to cease alertness and to ignore the walking human. Additionally, we examine the effect of direct gaze on distance variables, including the classical flight initiation distance (FID).

## Methods

### Ethical Statement

The research has been conducted according to relevant national and international guidelines.

### General

Experiments were conducted at 13 territories (8 in 2007 and 5 in 2008) of the Eurasian Magpies on the campus of Seoul National University, South Korea. The campus of Seoul National University represents a semi-urbanized environment where magpies are exposed to human pedestrians and are accustomed to human presence. The experiment was conducted on sunny days in mid-May 2007 and 2008, which corresponds to early nestling stage in the breeding of magpies. We conducted the experiments on the magpies that were not individually marked. However, magpies are highly territorial and immediately respond to any intruders by chasing them away. Thus, it is highly unlikely that any other bird than the breeder takes time to forage on the ground in the territory. We were aware of the boundaries of breeding territories, and we made sure that the testing sites were located well within the territory boundaries. Hence, we could be fairly sure that the foraging individuals were the territory owners. In early feeding period, the breeding female stays most of the time in the nest brooding the nestlings. Thus, we could easily observe one single magpie nearby the nest and we assumed that it was a male. We conducted experiments on these single magpies foraging near known active nests. Since the magpies were very accustomed to human presence, they did not show any vigilance behavior towards passing humans. The experimental procedure began after checking that the focal bird was continuously foraging for more than five minutes. We aimed at conducting at least a one gazing (up to four) and one non-gazing (up to five) experiment at each site in a randomized order. The order of experiments among the sites (gaze followed by non-gaze or non-gaze followed by gaze condition), and the dates of experiments for each site, were randomized within each year. For a given territory, the trials were done with at least two-day interval. Considering that the magpies see hundreds of people passing through their territories every day, and it is not uncommon to find people looking at magpies in the campus while walking, we do not think that habituation to the repeated testing might have occurred. In addition, the experimenters changed their clothes every day, and we believe that the experimental procedures were done in a manner that fully imitates the normal behavior of passers-by in the campus.

We use the term “gaze” for convenience to describe the situation where both the gaze *per se* and the facial direction are strictly correlated during the natural behavior of predators. Thus, in gaze condition, both eye gaze and facial direction were towards the bird; in non-gaze condition, both were away from the bird. In 2008, we carefully chose five experimental sites that were not examined in the previous year to avoid pseudo-replication (magpies are long-lived, stay in the same territories for many years and defend the territories year-round in our study area [49]).

We avoided situations with more than one magpie around because any social interaction between the magpies were likely to affect their responses to the experimental condition. We used the same walking speed of the experimenters across all the trials (approximately 1 step per second, which is similar to the pace of two people walking slowly together and talking to each other on the campus) and we conducted the experiments when there were no people around who can potentially affect the response of magpies. However, it is important to keep in my mind that numerous people frequently walked along those paths throughout the day and our experimental approaches were designed to imitate any such group of two young people walking on the path. In both years, the two walking experimenters were female students who performed the same role across the trials. The third experimenter, who recorded the trials in non-gaze condition (see below), were a female student in 2007 and a male student in 2008. None of the persons conducting the experiments took part in other research activities at the nests of magpies to assure that the birds do not individually recognize the experimenters [50].

### Type of Response and Timing

The main goal in our study was to measure the duration of the assessment time, which is the time between the moment of becoming alert and the moment of taking a behavioral action of a focal bird. The basic procedure was as follows. We selected the experimental sites based on the location of the pedestrian path and the lawn where the focal magpie was usually found foraging. When the magpie was approximately 1.5 m away from the pedestrian path (tangential distance) and about 15 m away from the experimenters, and there was no noticeable place for the magpies to hide, two experimenters started walking towards the foraging magpie while facing it. In ‘Gazing’ condition, both experimenters constantly looked at the foraging magpie. In ‘Non-gazing’ condition, both experimenters did not look at the magpie, and their behavior imitated typical human passers-by in the campus. One of the two experimenters who walked towards the magpie carried a stopwatch and measured the ***response time*** defined as the interval between the moment when the magpie showed the ***first response*** (i.e. recognition of our presence indicated by disruption of foraging and alert posture with the head up) and the moment when the magpie showed the ***second response***, which was categorized as one of the following: *ignore* (resume foraging), *walk away* (and eventually resume foraging at a distance to the path), *hop away* (and eventually resume foraging) or *fly away* (escape from human predator). The duration of hopping or walking away was not recorded, but it seemed that during that time the magpies did not stop watching humans. The other walking experimenter marked her own position on the path with the chalk bound to the tip of the umbrella at the moment when the magpie showed the second response, which marked the end of the experiment (for the estimation of distance variables, see below). In ‘Non-gazing’ condition when the walking experimenters could not directly and constantly observe the magpie, the ***response time*** and ***second response distance*** were measured from the video recorded by the third experimenter who filmed the experimental sessions from a 15–20 m distance (the walking experimenters and the focal magpie were visible in each video). From a sample of video recordings of gazing treatment, we confirmed that both methods of estimating the time and distance gave similar results.

### Distance Variables

**Figure 1:**
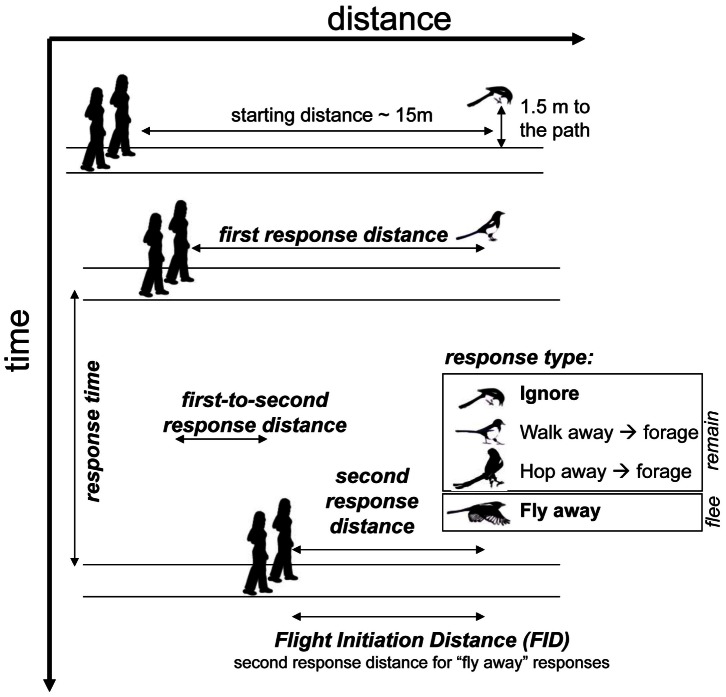
Schematics of the experimental methods and definition of variables.

Additionally we measured several distance variables, which are similar to variables already measured in other studies. We defined the distance between the experimenters and the location where the magpies showed the second response (***second response distance***). In 2008, we additionally measured the distance between the magpie and the two experimenters at the moment of the first response (***first response distance***). The distance variables are indices rather than absolute measures of distance between the human and the bird, because they were measured along the path of approach (Fig. 1), between the observer and the point of the shortest distance from the path to the bird’s initial location. Additionally, for those birds that hopped or walked away (Fig. 1), these distances are less accurate because unavoidably the bird moved away during the observers’ approach (but rarely more than by 1–2 m).

### Statistical Analyses

In order to examine the effect of gaze on the type of response of foraging magpies, we conducted multinomial regression on the four types of responses (*ignore*, *walk away*, *hop away* and *fly away*) with generalized linear mixed model. Identity of the foraging magpie (breeding territory identity, assumed from the proximity to bird’s nest) was treated as a random factor.

Because birds have a wide field of view [51]–[53] and because they might have only briefly looked strictly away from the moving experimenters, we believe that while walking or hopping away (Fig. 1; *walk away → forage* and *hop away→ forage*), the birds could still monitor the information from the approaching humans until the point where they resumed foraging. Therefore the time to the second response for these birds represented the duration of the risk assessment processes less precisely. But, the two extreme responses, *fly away* and *ignore*, more clearly showed that the bird abandoned careful (or any–in case of flying away) monitoring of the approaching human from the moment of decision to either forage again or to fly away. Therefore, for the main analysis, we only used the *fly away* and *ignore* responses to clearly determine whether gaze decreases the assessment time in prey (cognitive processing time) regardless of whether the approaching predator is assessed as dangerous (*fly away* response) or not (*ignore* response).

We used general linear mixed models to analyze the *response time* and *second response distance* (data collected in 2007 and 2008) as well as the *first response distance* (2008 data only) between gazing and non-gazing conditions, where the identity of the focal magpie was treated as a random factor. Year was included as an additional explanatory variable in all analyses. Throughout the text, averages values were given with standard errors. Our full dataset was unbalanced, and among our 13 magpies, 3 magpies were tested in only one treatment (gaze or non-gaze). In order to examine the robustness of our results, we repeated the same statistical procedures on 10 magpies that were tested in both treatments. Raw data can be delivered upon request. The analyses were conducted in SAS ver 9.3 (SAS Institute,Cary, USA).

## Results

### Type of Response and Timing

**Figure 2:**
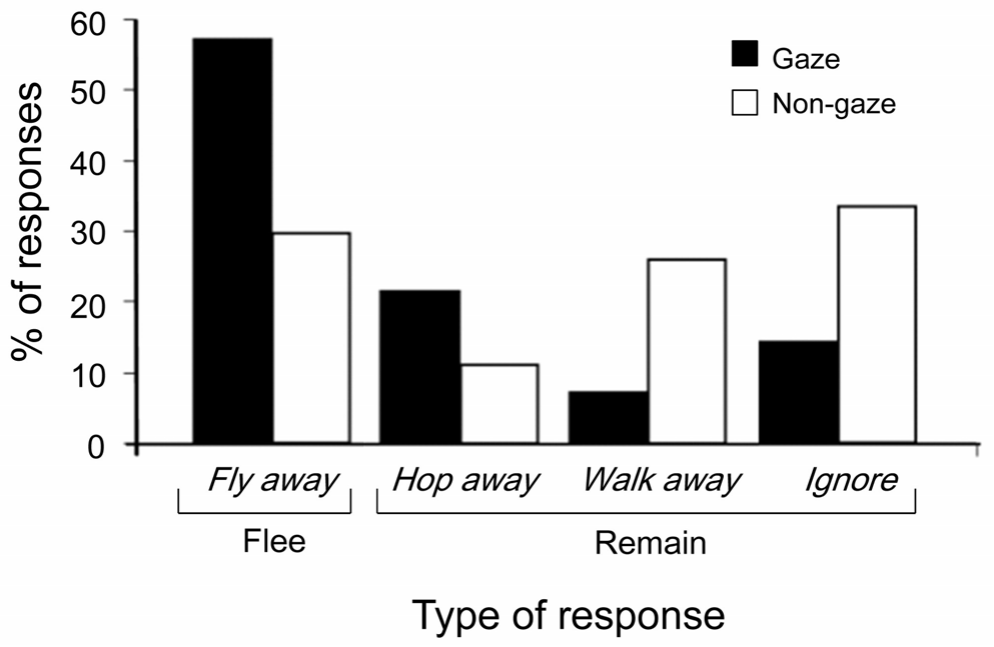
Effect of direct gaze on the frequency of second responses of 13 foraging magpies. 28 and 27 tests were conducted for gaze and non-gaze conditions respectively.

**Figure 3:**
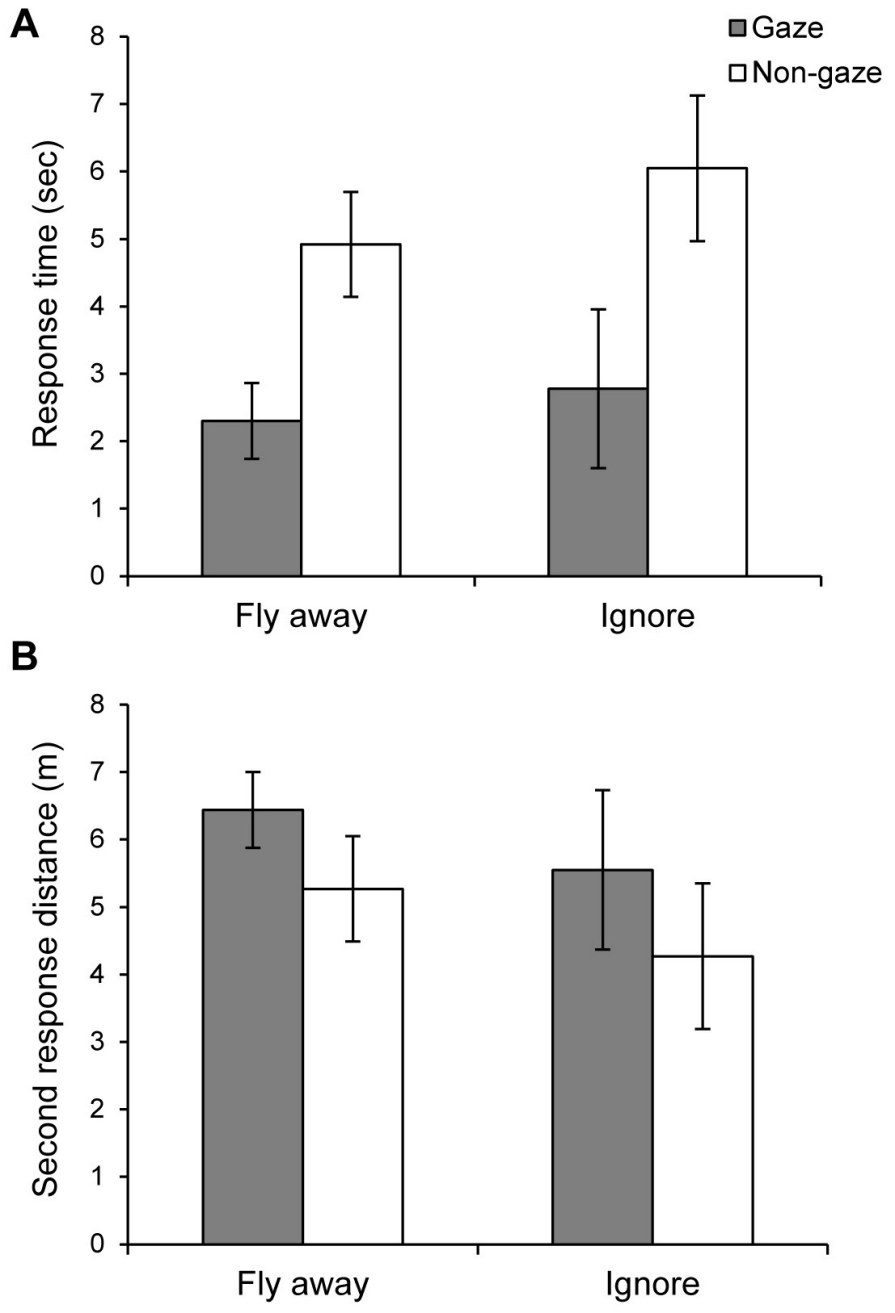
Effect of the direct gaze and the second response on the responses of 13 foraging magpies. (A) the effect of gaze on the response time. (B) the effect of gaze on the second response distance. Among the four types of second responses, *fly away* and *ignore* responses were compared. Grey bars represent data from gaze condition and white bars are for non-gaze condition.

The *second response type* differed significantly between gazing and non-gazing conditions (multinomial analysis, χ^2^ = 5.34, P = 0.021; Fig. 2). While magpies more often reacted by flying away in response to the gaze of experimenters, they more often resumed foraging or hopped/walked away from the path when the experimenters did not look directly at them. This indicates that humans, who walked along the standard campus paths and gazed directly at the birds foraging near the paths, were perceived by the birds as threatening more often than humans who did not gaze directly at the birds. However, regardless of the type of the second response, the *response time* was shorter when the experimenters looked directly at the birds: there was no significant interaction between the binary type of response (*fly away* or *ignore*) and the gaze treatment (gazing versus non-gazing), and there was no effect of the type of response (Fig. 3 (A), Table 1). Similar results were obtained when *hopping away* and *walking away* were pooled with *ignore* into one category “*remain*” and compared with the category “*flee*” (see Methods, Table S1 and Figure S1). This indicates that, regardless of the action taken by the bird in response to approaching humans, it took shorter time for the birds to initiate a behavioral action when humans directly looked at them, as if it was easier for the birds to estimate the degree of threat from an approaching human who directly gazed at them.

**Table 1:** The effect of gaze on the *response time* and the *second response distance* measured in 13 foraging magpies.

Effects	*Response time*		*Second response distance*	
	F_1,18_	Pr>F	F_1,18_	Pr>F
Gaze	11.29	0.002	2.01	0.174
Type of response	0.66	0.427	0.83	0.374
Gaze * type of response	0.13	0.720	0.00	0.953
Year	2.87	0.108	3.33	0.085

* Statistical results after removing the data from 3 magpies that were tested with either one of the treatments were qualitatively the same. The interaction between the gaze treatment and type of response was not significant for either *response time* (F_1,17_ = 0.12, P = 0.729) or *second response distance* (F_1,17_ = 0.21, P = 0.656). Similar to the results in the table, the effect of the treatment (i.e. gaze or non-gaze) was significant for *response time* (F_1,17_ = 10.48, P = 0.005) but not for *second response distance* (F_1,17_ = 1.69, P = 0.211).

### Distance Variables

All the distance-based variables showed trends consistent with previous studies of *FID* or with the trends observed in *response time*. The *first response distance* did not differ between gazing and non-gazing conditions (9.08±0.69 m in gazing condition and 9.53±0.81 m in non-gazing condition; F_1,18_ = 0.18, P = 0.68). This indicates that the distance at which the bird stopped foraging and became alert did not depend on the presence of gaze.

During the *response time,* the experimenter covered the *first-to-second-response distance* (estimated in 2008 only as *first response distance* minus *second response distance*). Because the *response time* was shorter in gaze condition, this distance was also shorter in gaze (3.84±0.47 m) than in non-gaze (5.18±0.55 m) conditions, albeit only marginally non-significantly so (F_1,18_ = 3.44, P = 0.08).

**Figure 4:**
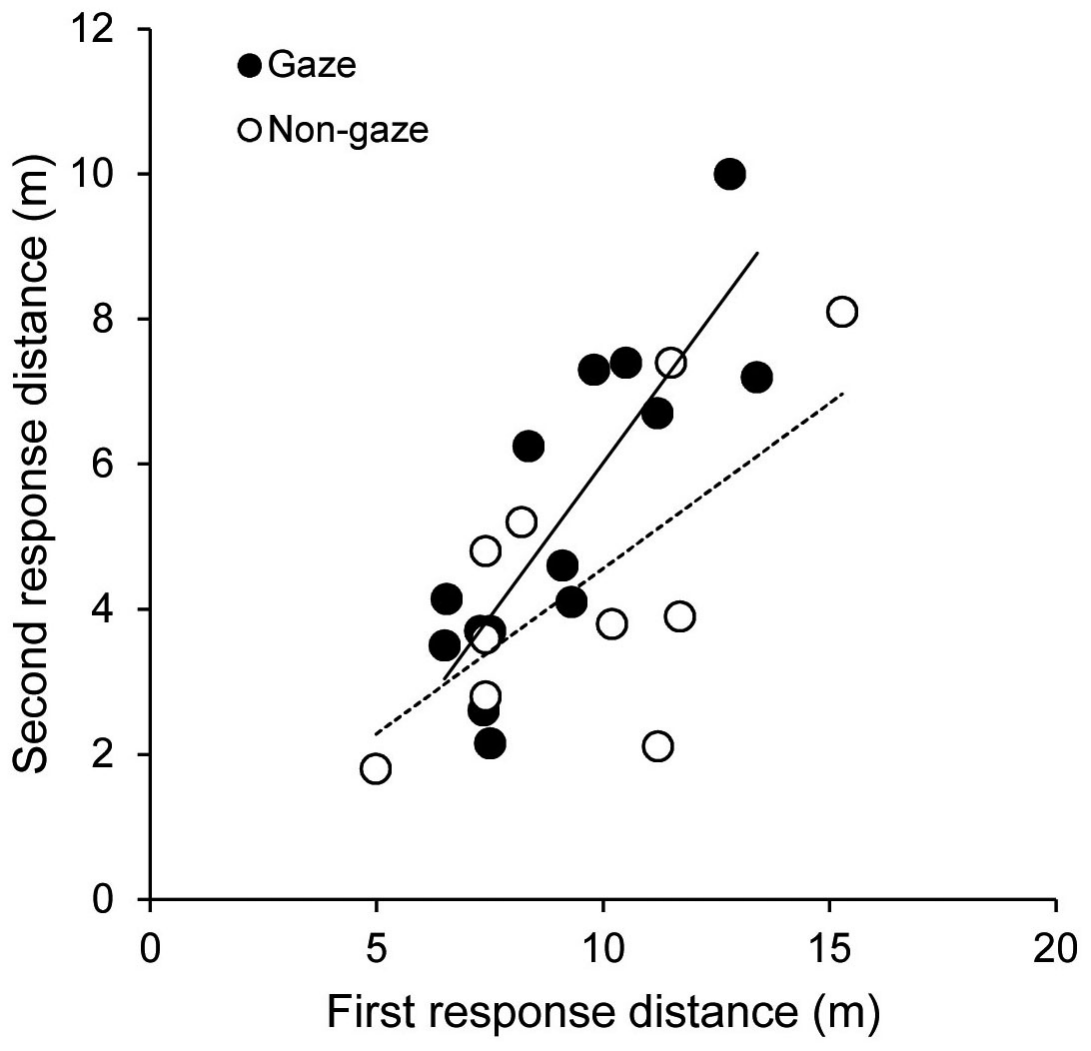
The relationship between the first response distance and the second response distance for gaze and non-gaze conditions.

Consequently, the *second response distance* was also affected. Although the *second response distance* did not differ significantly between experimental conditions in a simple analysis (Fig. 3 (B), Table 1), after including the *first response distance* as a covariate (for 2008 data only), the regression coefficient between *first response distance* (X) and the *second response distance* (Y) tended to be steeper in gazing (β = 0.85±0.18) than in non-gazing (β = 0.46±0.16) condition (interaction between the gazing type and *first response distance* is marginally non-significant; F_1,16_ = 2.67, P = 0.12; Fig. 4). All these trends in the distance variables appeared to be simple consequences of the difference in the *response time* between gazing and non-gazing treatments.

The *second response distance* for those birds that flew away (rather than resumed foraging with or without walking/hopping away to a new foraging spot) corresponds to the classical *Flight Initiation Distance* (*FID*; distance between predator and prey at the moment of prey escape initiation). In accordance with the classical literature, the second response distance tended to be larger in gazing (6.27±0.43 m) than in non-gazing (5.04±0.58 m) treatment but the effect was not significant (F_1,12_ = 1.34, P = 0.27).

## Discussion

We have shown here, for the first time, that the time to reach a decision and perform an action by a prey was shortened significantly when the predator directly looked at the prey, regardless of the type of prey’s reaction (to fly away or to ignore the predator approach), i.e. regardless of the perceived level of risk. These results suggest that, when an approaching predator directly looks at the prey, it reveals more information about itself and this extra information appears to be used by the prey to speed up the risk assessment process that leads to the choice of one of the several behavioral actions. The effect of direct look at the prey on the timing of prey behavioral response was independent from the effect of direct look on the level of perceived risk, because, similar to previous studies [39], [42], the birds flew away more frequently (a behavior indicating higher perceived threat) in response to humans looking directly at them.

We don’t know why a bird sometimes perceived the experimenters’ approach as risky and some other times not risky, but inter-individual and intra-individual variation in the risk assessment and decision making is expected [54]–[57]. None of the humans participating in the tests was familiar to the magpies therefore the effect of direct look (and exposing the face) cannot be explained by individual recognition, of which the magpies are capable [50]. However, we clearly showed that some features of a human who was directly looking at the bird caused the bird to respond sooner, indicating that the bird assessed the level of threat posed by the person faster when the person was directly looking at it. These features do not seem to directly indicate higher level of threat which was presented by the “direct look” of the person, because the shortening of the assessment duration (*response time* ***)*** was the same for the high-threat responses (fly away) and for the low-threat responses (remain) by prey. Gaze, face and head, as well as their movements, contain important clues that may allow to predict the actions of the subject [44]–[47], suggesting that they may also provide crucial information to the prey about the predator’s future actions. Hence, our results suggest that the effect of direct gaze (looking at the prey) of the predator on the behavior of the prey may extend beyond the classical issue of indicating the threat level [37]–[43]: the direct look from the predator may cause a general increase in information transmitted from the predator to the prey about the predator’s future actions.

Shortening of the assessment duration may be not only because of increased amount of information about the predators’ intent, but also because of increased level of stress. Stressors influencing the speed of decision may be viewed as serving an adaptive role by helping the animal to search for and scrutinize a source of danger [58]. We cannot entirely reject the possibility that stress was higher in the gaze condition for the birds who felt more threatened and chose to flee as well as for those who felt less threatened and chose to ignore, and that this enhanced attention of birds allowing all birds to come to a quicker decision irrespective of the nature of that decision. If this were true, then the behavioral response of flying away, while indicating higher level of perceived threat, would not have been a good indicator of higher level of stress. Future experiments that manipulate level of stress and threat separately may evaluate this hypothesis. Such a total disassociation between the level of stress and the level of threat was not possible in our situation of an approach of potentially threatening humans.

We designed our study to allow for a wide range of behavioral responses. Many experiments with human predators were designed to imitate the situation where no option for ceasing the alertness was given to the prey: the experimenters often moved along trajectories that were not clearly predictable from previous experiences of the prey with humans either because human presence was not common in the area, or because the experimenter would go along a straight line towards the prey across a terrain, ignoring paths along which normally humans move (e.g. [42], [59]). In our study, we used the system that is typical for most urban situations where animals are repeatedly exposed to humans walking along designated predictable paths, and we conducted experiments imitating the normal behavior of humans in the environment. Animals tend to be less threatened by movements of humans along standard paths [41], [60], [61], because the prey in this situation should not expect the predator to suddenly change its path (changing path affects the escape response in prey [31]). Hence, a bird near the path needs only to arrive at the assessment of whether it should fear or ignore the by-passer. The sheer effect of experience with humans abundantly present on the campus also increases the likelihood that some individuals will be less threatened [34], [42] and will perform behaviors other than escape. This situation is convenient for measuring the effect of various factors, such like predator’s gaze, on the duration of the assessment process that may result in both types of outcomes: flying away or ignoring the potential threat from the walking human.

From our results, we cannot discern whether magpies used the gaze itself or the face orientation as the cue, or whether they used other clues associated with a human looking directly at the bird. Recent studies attempted to tease apart the effect of gaze and that of the face orientation [62], [63]. However, we think that discriminating the effect of gaze from that of face orientation is largely irrelevant in typical predator-prey situations, because, unlike in subtle social situations [63]–[65], the gaze and the face direction of predators during their hunting behaviors are correlated such that there is no discrepancy between the gaze/look direction and the face/head direction [66]–[68]. The main predators of magpies in our population are the cats. Humans are the most common, although rarely lethal, threat. Avian predators are rarely found in our study area. Thus, we think that magpies in our study population developed keen alertness to the approaches and intentions of mammalian predators, and that they pay attention to the direction in which a predator looks and that direct look from the predator may, beyond indicating higher general risk, provide them with more detailed information useful in risk assessment and decision-making processes.

In summary, while our results were in agreement with the classical prediction of increased prey flight initiation distance (FID) in response to direct look from the predator, we have evidence that this effect may be present not only because the direct look indicates higher predation risk, as all previous papers assumed, but solely (or additionally) because the direct look makes the risk assessment faster for the prey. Most previous experiments always lead to the prey’s perception of high predation risk (sooner or later during the approach towards the prey the prey flew away from the predator). Therefore, they could not differentiate between the effect of direct look (or gaze) on lengthening of the FID because the direct look indicates higher perceived predation risk or because it facilitates faster risk assessment by the prey. The contribution of these two mechanisms can only be determined if experimental design promotes higher diversity in the perceived predation risk, and the behavioral response, by prey. We suggest that future studies in this area should use designs that allow distinguishing between the two mechanisms, and that theoretical models of optimal escape behaviors should incorporate the risk assessment duration, and factors affecting it, in their structure.

## Supporting Information

Figure S1 **Effect of the direct gaze and the second response on the responses of 13 foraging magpies; the effect of gaze on the response time (above) and on the second response distance (below).** “Flee” includes *fly away* responses, and “remain” includes *ignore, walk away* and *hop away* responses. Grey bars represent data from gaze condition and white bars are for non-gaze condition. Error bars denote standard errors.
(DOC)

Table S1 **The effect of gaze on the** ***response time*** **and the** ***second response distance*** **measured in 13 foraging magpies when the type of responses were coded as “flee (** ***fly away*** **)” and “remain (** ***ignore*** **,** ***walk away*** **, and** ***hop away*** **were pooled)”.**
(DOC)
