# Supplementary figures and images for: Direct Look from a Predator Shortens the Risk-Assessment Time by Prey
Source: PLoS One. 2013 Jun 5;8(6):e64977. doi: 10.1371/journal.pone.0064977 (PMC3673954; doi:10.1371/journal.pone.0064977)

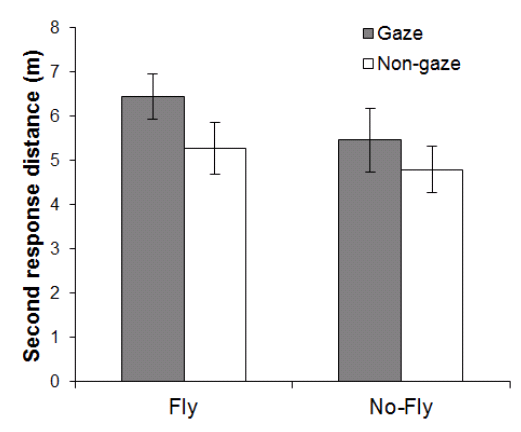


Flee

Remain


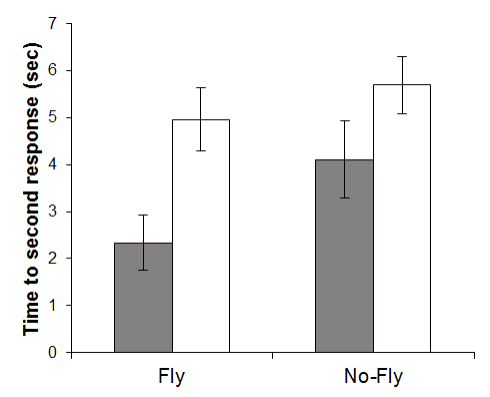


Flee

Remain

Supplement: Figure S1 — Effect of the direct gaze and the second response on the responses of 13 foraging magpies; the effect of gaze on the response time (above) and on the second response distance (below). “Flee” includes fly away responses, and “remain” includes ignore, walk away and hop away responses. Grey bars represent data from gaze condition and white bars are for non-gaze condition. Error bars denote standard errors. (DOC) [file pone.0064977.s001.doc]
