# Supplementary material for: Direct Look from a Predator Shortens the Risk-Assessment Time by Prey
Source: PLoS One. 2013 Jun 5;8(6):e64977. doi: 10.1371/journal.pone.0064977 (PMC3673954; doi:10.1371/journal.pone.0064977)
Supplement: Table S1 — The effect of gaze on the response time and the second response distance measured in 13 foraging magpies when the type of responses were coded as “flee ( fly away )” and “remain ( ignore , walk away , and hop away were pooled)”. (DOC) [file pone.0064977.s002.doc]

| **Effects** | ***Response time*** | | ***Second response distance*** | |
| --- | --- | --- | --- | --- |
|  | **F_1,46_** | **Pr > F** | **F_1,46_** | **Pr > F** |
| **Gaze** | **9.90** | **0.003** | 2.50 | 0.121 |
| **Type of responses** | 3.23 | 0.079 | 1.47 | 0.232 |
| **Gaze * type of responses** | 0.61 | 0.440 | 0.19 | 0.665 |
| **Year** | 3.63 | 0.063 | 7.77 | 0.008 |

66easured in 13 foraging magpies.led lity, one cannot infer one-tailed probability 000000000000000000000000000000000000000000000
